# Relationships between Social Resources and Healthful Behaviors across the Age Spectrum

**DOI:** 10.1155/2012/501072

**Published:** 2012-08-30

**Authors:** Kristina H. Lewis, Matthew W. Gillman, Mary L. Greaney, Elaine Puleo, Gary G. Bennett, Karen M. Emmons

**Affiliations:** ^1^Obesity Prevention Program, Department of Population Medicine, Harvard Medical School, Harvard Pilgrim Health Care Institute, Boston, MA 02215, USA; ^2^Department of Medical Oncology/Population Sciences, Dana-Farber Cancer Institute, Boston, MA, USA; ^3^Division of Biostatistics, Department of Public Health, University of Massachusetts, Amherst, MA 01003, USA; ^4^Department of Psychology and Neuroscience, Duke Global Health Institute, Duke University, Durham, NC 27708, USA

## Abstract

*Background*. We examined cross-sectional relationships of social resources with health behaviors in adults ages 18–93 years. *Methods*. Baseline data from a 2009 risk behavior intervention trial were used to measure social resources, physical activity, and fruit and vegetable intake in 2,440 adults. To evaluate associations overall and within 4 age groups (18–34, 35–49, 50–64, and 65–93 y), we used multivariable regression. *Results*. Mean (SD) age was 49.4 (15) years, physical activity was 346 (304) minutes/week, and fruit and vegetable intake was 3.4 (2.4) servings/day. Mean social resource score was 1.2 (0–4 scale) in 18–34 year olds, 1.1 in all other age groups (*P* = 0.04). In multivariable models, for each one-point increment in social resource score, the odds ratio for getting 150–959 minutes of physical activity/wk (compared to <150 min/wk) was 3.7 (95% CI 3.0–4.6). Each one-point increment in score was also associated with 29% (95% CI: 23–35%) more servings of fruit and vegetables. We did not observe effect modification by age group. *Conclusions*. Although younger adults reported slightly higher resources than older adults, the magnitude of association between social resources and healthful behaviors did not differ between them.

## 1. Introduction

Chronic illness causes much of the morbidity, mortality, and rising cost of medical care in the United States [1, 2]. Healthful behaviors, such as having a diet rich in fruits and vegetables and getting regular physical activity, can prevent or delay the onset and progression of chronic disease [3]. Higher fruit and vegetable intake is associated with decreased mortality from cardiovascular disease [4], prevention or delay of neurodegenerative disease [5], and improved cognitive function [6]. Physical activity decreases the risk of cardiovascular disease, cancer, and obesity [7] and is associated with decreased rates of incident dementia [8] and hip fracture [9, 10], as well as improved quality of life [11]. 

A substantial literature emphasizes the importance of the social and environmental determinants of healthful behaviors and health outcomes [12–20]. For instance, weight gain in a person's social network is associated with his or her own risk of obesity, through changes in behavioral norms and unconscious behavior imitation [21]. The frequency with which the elderly participate in social events correlates with their rate of motor decline [22]. Components of the neighborhood environment, such as walkability, access to parkland, and safety also influence personal health behaviors [12]. Even the work environment has an influence on health behaviors and outcomes, and employers are struggling to make their workers less sedentary through onsite wellness programs and other initiatives [23–25].

As adults age, their social and physical environments undergo a transformation. An exit from the working world, gradual physical and cognitive decline, and the loss of friends and loved ones may all contribute to smaller social and geographic circles [26, 27]. Further, the loss of structure and routines that accompanies retirement and household downsizing may reduce opportunities for social influence and personal commitment to healthy habits. Along with reducing access to social resources, a gradual decrease in financial flexibility or physical and mental well being could also reduce the ability of older adults to capitalize on such resources through decreased mobility or decreased ability to perform one's activities of daily living. For example, even if an 80-year-old woman receives advice on healthy recipes or sees an advertisement for a new fitness club in her neighborhood (both forms of social resources available to her), she might be less able to benefit from these resources than her 35-year-old healthy neighbor, because she does not do her own grocery shopping and she no longer drives a car. 

 In the aging literature, there is a growing emphasis on the idea of “aging in place” or allowing people to remain in their home environments for as long as they wish and are safely able to do so [28]. For aging in place to produce the best possible health outcomes and contribute to longevity, a clear understanding of the relationship between social resources and health behaviors across the age spectrum is necessary. It is possible that the shrinking geographic environment, changing nature of relationships, and physiologic transitions associated with aging may both decrease the availability of social resources and lessen the impact of resources such as active neighbors, a seniors group at the local health club, or family members who visit to share recipes for healthy dishes. 

The United States population is growing older [2], meaning that the burden of chronic illness will continue to rise. To address this problem, program developers and policy makers will benefit from understanding how social resources affect physical activity and dietary choices at different life stages. 

In this study of adults from across the age spectrum, we examined the relationship between social resources (from neighbors, friends, family, and local organizations) and the health behaviors of fruit and vegetable intake and physical activity. We hypothesized that social resources in older adults would be fewer than in younger adults and would have less correlation with physical activity and fruit and vegetable intake.

## 2. Methods

### 2.1. Study Design and Participants

Study participants were enrollees in “Healthy Directions 2,” a randomized trial of a multiple risk behavior intervention. We recruited participants at their annual well visit or chronic disease management appointment during 2009 in one of 2 urban primary care centers in the Boston area. Eligibility requirements included being 18 years or older and having the ability to read and write in English. Patients were excluded if they had received cancer therapy within the past year or carried a diagnosis of dementia or other neurodegenerative disorder or major psychiatric condition within the past 5 years. 

Participants were approached first with a mailing, then enrolled in person at a medical appointment. At that visit, enrollees completed a self-administered baseline questionnaire detailing their health habits, perceived level of social support for healthful behavior and demographics. We obtained participant age, sex, and primary care provider (PCP) identity from electronic medical records. Of all approached to participate in the trial, 52% (*n* = 2, 440) enrolled and completed the baseline survey. For our analyses, 123 participants were excluded due to incomplete data on the social resources measure, 185 for missing physical activity data, and 208 for missing fruit and vegetable intake data.

### 2.2. Main Measures

Our primary exposure measure was the level of social resources reported by participants in the baseline survey, as assessed by 3 subscales from the “Chronic Illness Resources Survey” (CIRS). The original CIRS was a 22-item instrument used to evaluate support for chronic illnesses and healthful behaviors. This instrument was designed to be adapted and modified for other populations or research questions [29]. The CIRS includes a number of support resources that reflect a range of processes by which social support may influence behavior, including engaging together in a specific behavior and providing support or actions that facilitate a behavior. The purpose of using the CIRS in this context was to determine the extent to which social resources are effective promoters for the target behaviors. Our survey for this study included the family and friends, neighborhood, and organizational subscales from the original CIRS, for a total of 9 items, each measured on a 5-point scale (ranging from 0, not at all to 4, very often). The specific items we used are provided verbatim in Table 1 of this paper. 

We summed the points associated with each participant's responses and averaged by their number of completed items, to yield a total CIRS score of 0–4 points. We excluded participants who answered fewer than 7 of the 9 items (*n* = 123). Because socioeconomic position may be intricately linked to social resources, we did assess this as well (described below under other measures).

There were two outcome measures in this study—weekly minutes of moderate and vigorous physical activity and daily servings of fruit and vegetables. We assessed physical activity using four questions adapted from CDC's Behavioral Risk Factor Surveillance Survey (BRFSS) [30], which included descriptions of moderate (e.g., brisk walking, biking, or anything that causes small increases in breathing or heart rate) and vigorous (e.g., running, aerobics, or anything else that causes large increases in breathing or heart rate) activities. We summed reported moderate and vigorous physical activity into a total number of weekly minutes and assigned each participant to one of 3 categories, less than 150, 150–959, or 960 or more minutes per week. We set the lower cutoff at 150 minutes because that is the minimum weekly amount of moderate and vigorous physical activity recommended by the CDC [31]. We chose 960 minutes per week (corresponding to just over 2 hours per day) as the upper cutoff, as suggested in the literature [32], and because we felt this group of respondents were extreme in their level of reported physical activity. 

We assessed fruit and vegetable intake using the National Cancer Institute's “5 A Day for Better Health” tool, a 7-item instrument assessing frequency of intake for orange juice, other fruit juices, fruit, green salad, French fried potatoes, other kinds of potatoes, and other vegetables [33]. Using the responses to 6 of the 7 items (we excluded French fried potatoes), we calculated a total number of daily servings of fruits and vegetables for each participant. 

### 2.3. Other Measures

Covariates from the medical record included sex and age, with age at enrollment categorized into the following groups: 18–34, 35–49, 50–64, or 65 years or older. We used survey data to obtain information on race/ethnicity (white, black, Hispanic, Asian, or other) and education (did not graduate from high school, high school graduate/GED, some college or 2-year degree, or 4-year college degree or more). We assessed financial situation by having participants rate the money situation in their household (“comfortable with extras,” “enough but no extras,” “have to cut back,” or “cannot make ends meet”), which, as opposed to household income, minimized missing data and is relevant for use in retirement-age participants. Participants also reported current weight and height from which we calculated body mass index (BMI) using the formula weight (kg) divided by the square of height (m). Self-reported health status was determined using the one-item assessment from the Medical Outcomes Study Short-Form [34]. We also included a covariate corresponding to the PCP ID for each participant.

### 2.4. Statistical Analysis

We first examined the bivariate associations between age group and physical activity and fruit and vegetable intake. We also examined how CIRS score (and subscales) varied by age. We then used regression models to evaluate the relationship between CIRS score and the two outcomes. We modeled the relationship between CIRS and physical activity using multinomial logistic regression among the 2,155 participants with data on these 2 variables and the CIRS and fruit and vegetable intake relationship using negative binomial regression among the 2,134 participants with data on these 2 variables. Rather than reporting the negative binomial regression estimate as the log odds of changing fruit and vegetable intake, we calculated the antilog of the parameter estimate to show the predicted change in the ratio of fruit and vegetable intake per one point increment in CIRS score. We created multivariable models adjusted for sex, age group, race/ethnicity, education, financial status, BMI category, self-reported health status, and primary care provider. To assess whether age group had a modifying effect on the relationship between social support and healthful behaviors, we included interaction terms for “age group × CIRS score” in each of our final models. We conducted all analyses using SAS, V.9.2. (Cary, North Carolina). 

## 3. Results

### 3.1. Population Characteristics

The majority of participants (65.7%) were women, with a mean (SD) age of all participants at baseline of 49.4 (15) years, ranging from 18 to 93 (Table 2). Just over half (54.7%) of participants were white. Sixty percent had a college degree, and about 46% rated their financial status as comfortable. Mean (SD) BMI was 28.1 (6.2) kg/m^2^, and participants were about evenly split between the normal, overweight, and obese BMI ranges. Slightly over one-half (56%) rated their health as very good or excellent.

**Figure 1:**
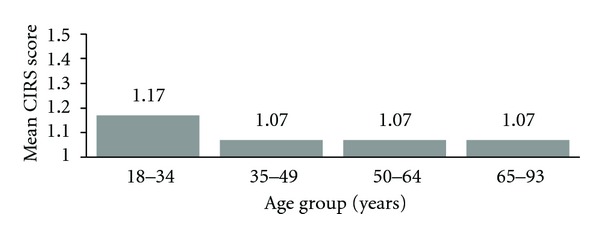
Mean score on 9 items of the CIRS (0–4 point scale) according to age group. one-way ANOVA *P* value across age groups = 0.04.

The mean CIRS score was 1.09 (SD 0.64, range 0–4). The youngest participants (age 18–35) reported slightly higher levels of social resources (mean 1.17) than those in the older age groups (mean 1.06) (one-way ANOVA *P* = 0.04) (Figure 1). Among the 3 CIRS subscales, only the level of family and friends support differed by age: the score was 1.32 in the youngest age group, 1.21 among 35–49 year olds, 1.26 among 50–64 year olds, and 1.10 among the 65–93 year olds (one-way ANOVA *P* = .0005). 

**Figure 2:**
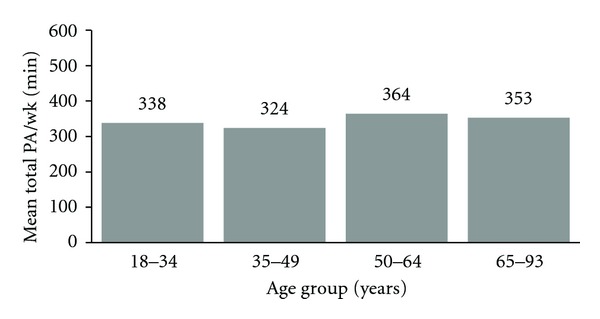
Self-reported mean moderate and vigorous physical activity, according to age group. One-way ANOVA *P* value across age groups = 0.07.

**Figure 3:**
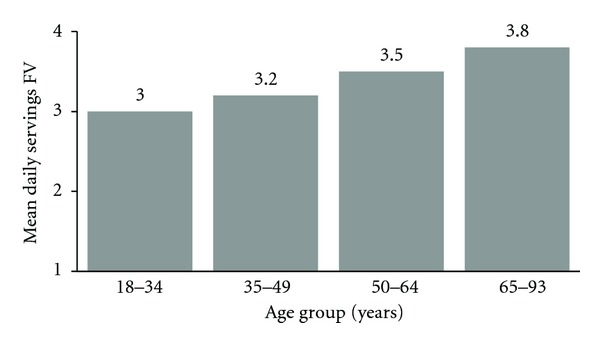
Self-reported mean fruit and vegetable intake, according to age group. one-way ANOVA *P*-value across age groups < 0.0001.

Mean SD reported moderate and vigorous physical activity was 346 (304) minutes per week (Table 2). Reported PA levels did not differ substantially across age groups (Figure 2), with those 65 years and older actually reporting a mean of 353 minutes weekly and a one-way ANOVA test showing no significant variability across age groups (p 0.07). Participants reported a mean (SD) intake of 3.4 (2.5) servings of fruits and vegetables per day, with increasing mean number of servings by age group, such that the oldest adults reported 0.8 more servings per day (3.8) than the youngest (3.0) (one-way ANOVA *P* < 0.0001) (Figure 3).

### 3.2. Relationships between Social Resources and Healthful Behaviors

The crude multinomial logistic regression model of the relationship between CIRS score and physical activity showed that each one-point increment in CIRS score was associated with greater odds of reporting higher physical activity levels (relative to reporting <150 minutes/wk) (Table 3). Specifically, the OR (95% CI) for reporting 150–959 minutes of moderate and vigorous physical activity per week was 4.1 (3.4–4.9), and it was 4.4 (3.4–5.8) for reporting ≥960 min/wk. The results of multivariable models were similar to those in the crude models. The fully adjusted multinomial regression model for the CIRS physical activity relationship also showed increasing odds of higher physical activity for each one-point increment in CIRS score (3.7 (3.0–4.6) for 150–959 min/wk and 4.7 (3.5–6.3) for ≥960 min/wk, compared to <150 min/wk) (Table 3). 

The crude negative binomial regression model of the CIRS fruit and vegetable relationship showed that each one-point increment in CIRS score was associated with 34% (95%CI 28–40%) more daily fruit and vegetable intake (Table 3). When this model was adjusted for our covariates, we found little change in the outcome, with the adjusted percent increase in fruit and vegetable servings at 29% (95% CI 23–35%) for each one-point increment in CIRS score (Table 3).

In order to test for effect modification by age, interaction terms for “age group × CIRS score” were introduced into the models. In the case of the physical activity outcome, the interaction terms were nonsignificant, with *P* 0.8 for 150–969 min/wk and *P* 0.9 for ≥960 min/week. For fruits and vegetables, the interaction terms were in ascending order of age group: 0.18, 0.95, 0.97 (>65 year olds as comparison group). This was also indicative of a lack of effect modification of the resource-behavior relationship by age group. 

## 4. Discussion

In this large study with participants representing a wide age range and considerable racial/ethnic diversity, we found that greater social resources correlated with higher levels of physical activity and higher fruit and vegetable intake in adults of all age groups. Levels of social resources were slightly higher among younger participants, mainly due to greater support from friends and family, but, contrary to our hypothesis, older age did not appear to lessen the impact of social resources on behavior. 

Although few data exist on how to interpret the CIRS score, our mean score of 1.09 was lower than the mean CIRS score of 2.7 previously reported in work by Glasgow et al. among a group of older women with diabetes [29]. Their score of 2.7, however, was based on the complete 22-item CIRS instrument, and in that study the average scores for the 3 subscales used here were also lower than for other CIRS subscales measured (family and friends 2.37, neighborhood 2.14, organizational 1.71) [29]. Even accounting for that difference, our subscale scores seemed low, a surprising finding given that a large percentage of our group was highly educated and financially comfortable [35].

The slightly higher CIRS scores in younger participants within this study were due to higher levels of resources from family and friends, which may reflect that older adults in our sample have fewer social contacts or that these family members and friends were simply less likely to encourage things like physical activity and fruit and vegetable consumption in older persons. Although statistically significant, the clinical impact of these different levels of social resources is unclear, given that adults 65 and older were just as likely as young adults to be physically active and more likely to meet recommended levels of fruit and vegetable intake. 

The participants in our study reported high levels of physical activity, but, when viewed categorically (less than 150 min/wk versus more than 150 min/wk), these numbers may be on par with national samples relying on self-reported information. The 2007 nationwide BRFSS, for example, estimated that 60.4% of women and 68.9% of men met recommendations for “regular physical activity” [36]. In our sample, 67% of respondents reported at least 150 minutes per week, with many reporting substantially higher levels. Unlike the BRFSS nationwide sample, we did not find that weekly physical activity decreased with age [36]. When viewed as a continuous variable, our participants reported a mean of 346 minutes of total (moderate + vigorous) physical activity per week. The “Walk Kansas” study by Estabrooks et al. relied on the same items we used to assess physical activity, and within their most active group at baseline, the mean total (moderate + vigorous) physical activity reported was 308 minutes/week, not substantially different from our overall mean [30]. To guard against bias from potential overreporting, we analyzed physical activity as a categorical rather than continuous variable.

Study participants reported eating an average of 3.4 servings of fruits and vegetables daily, less than the recommended 5 servings, but consistent with previous research on adult patterns of fruit and vegetable intake in the US [37–39]. Older adults in our study reported higher levels of fruit and vegetable intake than younger adults. This finding could be due to unmeasured factors such as older adults eating more meals cooked at home [40] or to other diet-related lifestyle choices that may differ between age groups. 

The direct relationship we observed between the social resource score and physical activity was consistent with our expectations. Previous work by Glasgow has shown a high degree of correlation between CIRS scores and physical activity levels [29]. The strength of this relationship could have a lot to do with how social resources are being measured in the instrument. For example, a 2009 systematic review concluded that subjective evaluations of social resources had greater correlation with physical activity than objective measures [27]. Other instruments using subjective measures of social resources have also shown strong links to physical activity behavior. For example, a 2003 cross-sectional study of Australian adults found that higher levels of subjective social resources from friends and family and membership in organizations that promote physical activity did correlate with higher levels of walking [41]. 

The direct relationship between social resources and fruit and vegetable intake has also been explored in the literature among different populations [18, 42–44]. Relating to our study, Glasgow and colleagues studied the link between CIRS scores and dietary behaviors and found that there was a high degree of correlation between the two and that changes in CIRS score predicted changes in behaviors in the setting of a longitudinal study [29]. The findings in our cross-sectional study support those noted by Glasgow et al. but our population is novel in that it includes people from across the age spectrum, showing that influences at the family and friends, neighborhood, and organizational levels directly correlate with increased fruit and vegetable intake behavior in adults of all ages.

We questioned whether the relationship between social resources and health behaviors would be different between older and younger adults and found that these relationships did not vary by age. Our hypothesis was based on the idea that older adults may be less able to capitalize on available resources than younger adults, due to physical, financial, and other limitations. Although other papers have examined the social support-behavior relationship exclusively in older adults [16] or compared its effects across racial/ethnic groups [17], this particular research question has not been previously explored in any paper of which we are aware. The lack of effect modification we observed suggests that, regardless of age, improved social resources from family and friends, neighborhood, and local organizations correlate equally with more healthful behavior. This could be because social resources act through different, but equally effective, mechanisms in the different age groups. For example, it could be that older adults are more likely to take advice and support from their offspring, whereas younger adults are more likely to achieve behavior change due to pressures from friends and colleagues. Furthermore, the relationship between social resources and behavior is complex and likely modified by factors that were unmeasured in our study, such as features of the built environment and self-efficacy [16]. However, our findings could also be due to a relatively small number of the “oldest old” within our sample. Although we did enroll participants as old as 93 years, those of advanced age were relatively few (only about 5% of those 65 and up were 85 years or older). 

## 5. Limitations

Our study was cross-sectional, limiting causal inference. As is typical of large population level studies, most measures were based on self-report, which can lead to misclassification of exposure or outcome. As we would not expect the misclassification in this case to be differential, our estimates of effect are probably either closer to the null or associated with larger standard errors, both conservative biases. Residual confounding is possible in any observational study, but the relative lack of attenuation from crude to multivariable models argues against it as a major threat to validity. With regard to physical activity, we assessed only moderate and vigorous activity. Though we asked participants to include brisk walking in their estimates of physical activity, we did not analyze it separately, which could have added to the strength of our measure. Additionally, although we had an overall measure of health status, we did not specifically assess functional mobility of the participants or have an exact measure of ability to perform ADLs. Finally, our analyses did not examine for possible three-way interactions between things like race/ethnicity or gender, with age and social resources. This could potentially have masked underlying differences between races or between men and women with respect to the aging-social resources-health behaviors continuum. 

Although our cohort is diverse in terms of race/ethnicity and age, the participants were also generally financially comfortable and well educated, which may limit the generalizability of our findings. 

## 6. Conclusions

As the US population ages, the burden of chronic illness will undoubtedly increase. Although cross-sectional, our findings support the hypothesis that higher levels of social resources at the family, neighborhood, and organizational levels are associated with higher physical activity and fruit and vegetable intake in older as well as younger adults. Longitudinal studies and interventions are necessary to understand how programs and policies to bolster social resources can result in improved health-related behaviors and outcomes. Supporting such social resources may promote longevity and independence and decrease the need for costly medical services.

## Figures and Tables

**Table 1:** Items from the CIRS used in our baseline survey* [29].

CIRS subscale identity	Items used
Family and friends	“Family members or friends exercised with you”
	“You shared healthy low-fat recipes with friends or family members”
	“Family or friends bought foods or prepared foods for you that were especially healthy or recommended”

Neighborhood	“You walked or exercised outdoors in your neighborhood”
	“You ate at a restaurant that offered a variety of tasty, low-fat food choices”
	“You walked or did other exercise activities with neighbors”

Organizational	“You attended free or low-cost meetings (e.g., walking groups/clubs, Weight Watchers, church groups, to support healthy habits”)
	“You volunteered your time for local organizations or causes”
	“You attended wellness programs or fitness facilities (e.g., the YMCA, Reggie Lewis Center, Gold's Gym, or Total Fitness)”

*Items were preceded with the following prompt: “For each question, please mark the response that best indicates your experience over the past 6 months.” Participants could then choose to mark one of the following options: “Not at all,” “A little,” “A moderate amount,” “Quite a bit,” or “Very often.”

**Table 2:** Characteristics of participants (*n* = 2, 440).

Characteristic (*N* respondents)	*N* (%)
Sex (2,440)	
Female	1603 (65.7%)
Age (years) (2,440)	
18–34	494 (20.2%)
35–49	679 (27.8%)
50–64	921 (37.8%)
≥65	345 (14.1%)
Race/Ethnicity (2,350)	
White	1307 (54.7%)
Black	667 (27.9%)
Hispanic	215 (9%)
Asian	107 (4.5%)
Other	54 (2.2%)
Education (2,403)	
Less than high school	89 (3.7%)
High school graduate/GED	296 (12.3%)
Some college or 2-year degree	577 (24%)
≥4-year college degree	1441 (60%)
Money situation in household (2,367)	
“Comfortable with extras”	1083 (45.8%)
“Enough but no extras”	683 (28.9%)
“Have to cut back”	476 (20.1%)
“Cannot make ends meet”	125 (5.3%)
BMI (kg/m^2^) (2,327)	
Normal (18.5–24.9)	771 (33.1%)
Overweight (25–29.9)	785 (33.7%)
Obese (≥30)	771 (33.1%)
Health status (2,440)	
Very good/excellent	1282 (52.5%)
Good	860 (35.2%)
Fair/poor	298 (12.2%)
Moderate and vigorous physical activity category (2255)	
<150 min/wk	735 (32.6%)
150–959 min/wk	1289 (57.2%)
≥960 min/wk	231 (10.2%)

Fruit and vegetable intake (servings/day) (2232)	Mean (SD)

	3.4 (2.5)
CIRS scores (0–4 scale) (2317)	
Overall	1.09 (0.64)
Family and friends	1.19 (0.91)
Neighborhood	1.36 (0.83)
Organizational	0.74 (0.78)

**Table 3:** Associations of score on 9 items of the CIRS (0–4 scale) with self-reported moderate and vigorous physical activity and self-reported fruit and vegetable intake.

Moderate and vigorous physical activity category (min/wk)	*N*	Crude	Adjusted
		OR (95% CI) for one-point increment in CIRS score*	

<150	707	1.0 (Ref)	1.0 (Ref)
150–959	1233	4.1 (3.4, 4.9)	3.7 (3.0, 4.6)
≥960	215	4.4 (3.4, 5.8)	4.7 (3.5, 6.3)

Fruit and vegetable intake count data		Expected ratio of increase in FVI count (servings/day)	
		(95% CI) per one-point increment in CIRS score**	
	2134	1.34 (1.28, 1.40)	1.29 (1.23, 1.35)

*Multinomial logistic model adjusted for sex, age, health, education, financial situation, race/ethnicity, primary care provider, and BMI.

**Negative binomial model adjusted for sex, age, health, education, financial situation, race/ethnicity, primary care provider, and BMI.
